# Directive Multiband Antenna Based on Rectangular Loop Array and Dumbbell-Shaped Slot Radiator

**DOI:** 10.3390/s21217082

**Published:** 2021-10-26

**Authors:** Jose Alfredo Tirado-Mendez, Hildeberto Jardon-Aguilar, Ruben Flores-Leal, Luis Alberto Vasquez-Toledo, Arturo Rangel-Merino, Rricardo Marcelin-Jimenez, Enrique Rodriguez-Colina, Michael Pascoe-Chalke

**Affiliations:** 1 Instituto Politécnico Nacional, SEPI, ESIME-Zacatenco, Av. IPN S/N, U.P. Adolfo Lopez Mateos, Mexico City 07738, Mexico; arangelm@ipn.mx; 2 UAM-Iztapalapa, Electrical Department, Av. San Rafael Atlixco 186, Leyes de Reforma 1ra Secc, Iztapalapa, Mexico City 09340, Mexico; lvasquezt@xanum.uam.mx (L.A.V.-T.); calu@xanum.uam.mx (R.M.-J.); erod@xanum.uam.mx (E.R.-C.); mpascoe@xanum.uam.mx (M.P.-C.); 3 Telecommunications Section, CINVESTAV-IPN, Av. IPN 2508, San Pedro Zacatenco, Mexico City 07360, Mexico; hjardon@cinvestav.mx (H.J.-A.); rfleal@cinvestav.mx (R.F.-L.)

**Keywords:** resonant loop, slot radiator, directive antenna, multiband antenna, antenna array

## Abstract

In this article, a combination of rectangular loop array and slot radiator for multiband applications is presented. The antenna is configured by arranging, concentrically, a set of rectangular loop radiators excited by electromagnetic coupling provided by a dumbbell slot. The size of the loops is calculated to obtain the desired resonant frequencies, which are almost independent of the adjacent rings. The exciting slot is designed to operate in a wideband frequency range to cover the upper desired resonance. In addition, to obtain directive radiation patterns, a reflector shaped like a box is introduced, giving a stable gain, radiation pattern shape, and port matching at the selected frequencies. The configuration presents great results, since to the authors’ knowledge, even a similar configuration given in the open literature presents some disadvantages compared to this one; moreover, not just any structure can be employed as the resonating elements, obtaining multiband behavior at the same time.

## 1. Introduction

Current communications systems require the use of several bands to provide different types of services, such as Wi-Fi, telephony, GPS, and Bluetooth, among others. According to these requirements, systems need different antennas to support such necessities. These elements can be designed to perform over a wide bandwidth if the system is limited to just one antenna. The other way is to utilize multiband antennas. The difference between using wide bandwidth or multiband antennas is related to electromagnetic compatibility concerns.

Some of the techniques applied to achieve this goal are the implementation of fractal configurations [1], where each fractal iteration leads to a different resonance of the antenna and in some cases to generating a wideband operation. On the other hand, there are other methods to obtain the multi-band performance, such as metamaterial [2], electromagnetic band-gap structures (EBG) [3], or defected microstrip structures [4]. Regarding the latter, the goal is to modify the current distribution of the radiator and generate an extra-inductance, which at the same time, combined with the associated capacitance of the radiator, generates a new resonance, commonly, at a lower frequency compared to that provided by the antenna with no extra technique.

It is noteworthy that most of these techniques are exploited in planar antennas developed on substrates. These antennas have many advantages, but one of their biggest drawbacks is their limited applications in high power signals.

The use of loop antennas has been employed for many years, and some works have described the proper operation of this kind of radiator [5,6] for a circular geometry. Some recent publications demonstrate the use of several techniques to create frequency reconfigurable antennas, such as slot-lines [7,8], annular rings [9], split-ring resonators [10,11], or even the complement of a slot-ring combination [12,13]. However, these approaches carry out the excitation of a few resonances, reaching no more than three useful bands. In [14], a description of a square loop is developed, and the operation and electromagnetic field’s closed-form expressions are obtained. In [15], authors proposed a combination of concentric square rings to generate a multiband antenna. This antenna is excited by a combination of a microstrip feeding and a slot for electromagnetic coupling. However, there are some disadvantages in that work regarding higher modes excitation, undesired resonances, the use of several slots, one for each square, and other features that will be pointed out in the following sections.

Alternatively, slot antennas have also widely been utilized either as radiator [16], as well as a reactive load to enhance the performance of an antenna [17]. One important advantage of designing slot antennas is its simplicity. Well described methods for the slot design have been reported [18,19], where expressions are given to obtain the theoretical dimensions of the slot to operate at a certain frequency.

Combining those methods, for slot and loop radiator, the next section shows the design of the proposed multiband antenna. In this article, a multiband antenna with the characteristic to select all or some of the required frequencies is implemented by using a set of concentric rectangular loop resonators. The loop array is excited electromagnetically by a circular-dumbbell slot radiator; thus, with this configuration, a compact and multifunctional antenna is obtained. The antenna can be designed to operate with a bidirectional or directive radiation pattern, according to the requirements.

The next sections show the steps in the design of the radiating slot excited by a coaxial cable, the design and configuration of rectangular loop radiators, as well as the proposed configuration and, finally, the construction and measurements. The antenna prototype presents resonances at 1.71, 1.84, 2.0, 2.22, 2.48, 2.81, 3.33 and 4.92 GHz, with gain levels going from almost 4 to 8 dB with a steady directive-shaped radiation pattern. The average bandwidth in each resonance is around 30 MHz at the lower frequencies, and more than 100 MHz at the higher resonances.

## 2. Antenna Design and Simulations

To demonstrate the process, the combination of rectangular loops and the slot radiators is presented for arbitrary resonant frequencies, taking into account a large number of resonances. However, the configuration is not limited by these requirements, since for specific resonances, the method is also valid and effective just by considering the number of bands that must be achieved, even if this number is small.

*A*. *Slot radiator design*

The first step is to design the radiating slot. The slot is made on a thin metal sheet and is designed to operate at a frequency of 5 GHz. This frequency was selected since it is higher than the resonances that will be obtained when the rectangular loops are incorporated. One important thing that must be taken into account is that if the highest frequency required is $f_{1}$, then the design must start choosing a resonant frequency of the slot $f_{\mathrm{slot}}$ higher than $f_{1}$, since the new resonant frequencies are lower than $f_{1}$ when the rectangular loops are integrated to the slot due to the reactive load generated by the added structures.

Then, to not overlap the resonances provided by the slot and by the loops, a high value must be considered. For a slot radiator built on a substrate with dielectric permittivity εr, the expression is given by (1) [18].

(1) $$\lambda_{S}/\lambda_{e}=1.045-0.365\ln\varepsilon_{r}+\frac{6.3\left(\frac{W}{h}\right)\varepsilon_{r}^{0.945}}{238.64+\frac{100W}{h}}-\left[0.148-\frac{8.81\left(\varepsilon_{r}+0.95\right)}{100\varepsilon_{r}}\right]\ln\left(h/\lambda_{e}\right)$$

where $\lambda_{e}$ is the average wavelength given by the propagation in the free space and the interaction of the substrate, and $\lambda_{S}$ is the wavelength at the slot; W and h are the slot width and substrate thickness, respectively. In this case, the slot design is much simpler since no substrate is implemented, because the radiator is developed over a metal sheet. Taking into account this consideration, a radiating slot length with no substrate can be approximated by the wavelength at free space. Considering a slot length of λ, the dimension is 60 mm at 5 GHz. This length is chosen since the slot can resonate with this dimension or even submultiples, such as ½, 1/8, or including 1/16, but with one wavelength, many resonances can be added by including several loops. The slot width was selected in order to get the antenna impedance close to 50 Ω when the input is taken in the middle of the slot length. However, as observed in (1), the electric length of the slot is dependent of its width, W; therefore, to initiate the design, the slot length is set to 60 mm and a parametric study was done using HFSS to determine the better slot width, considering bandwidth and port matching.

**Figure 1:**
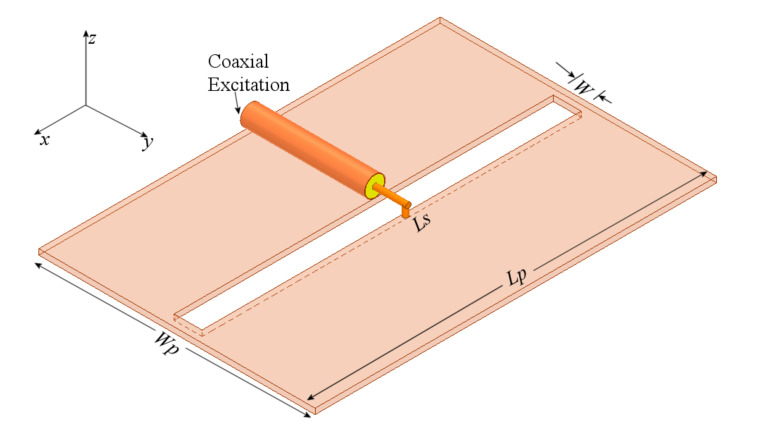
Slot radiator excited by a coaxial line.

**Figure 2:**
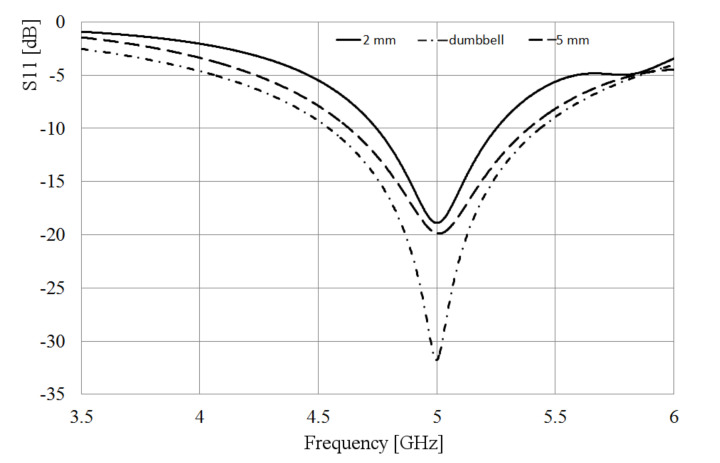
Simulated S11 parameter of the slot for different widths.

The slot is excited by a coaxial line as depicted in Figure 1, where the inner conductor of the coaxial cable is welded on one side of the slot, and the shielding conductor is soldered on the other side of the radiator. The metal sheet where the slot is etched has dimensions Lp = 64 mm and Wp = 49 mm and 1 mm thick. Bearing in mind two values of the slot width, W = 5 and 2 mm, after the simulation analysis, as expected, the slot length needed to be adjusted to obtain the required resonant frequency, changing from 60 mm to 68 mm for the wider slot, and 60 to 65 mm for the narrower slot. Figure 2 shows the simulated S11-parameter for both width (W) values.

**Figure 3:**
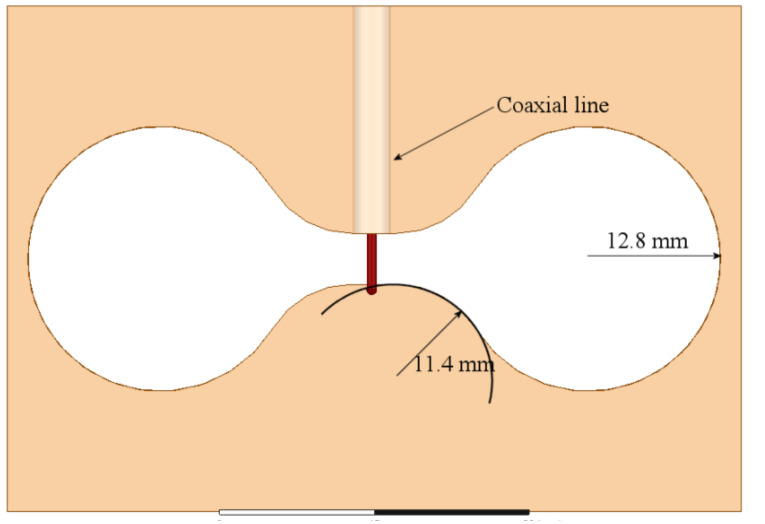
Modification of the rectangular slot to a circular dumbbell shape.

As observed in Figure 2, the matching and the bandwidth increase when the slot is wider. Following this idea, at the feeding point, the slot is kept 5 mm wide, but applying the Kraus technique [20] to the remaining arms of the slot, they are broadened circularly, forming the shape presented in Figure 3. The length of the slot is also kept to 68 mm. The rest of the dimensions shown in Figure 3 are optimized by using HFSS, looking up to better the bandwidth and the port matching.

To compare the performance of the dumbbell-shaped slot to the rectangular one, the simulated S11 parameter is also presented in Figure 2. As displayed in this figure, the slot bandwidth goes from 4.5 GHz to 5.4 GHz, almost 1 GHz, with a deeper port matching, reaching a value below −30 dB. The idea behind achieving a wider bandwidth with the slot is, mainly, to overcome the disadvantage of shifting the resonance of the antenna to an out of band frequency when the loop radiators are integrated to the slot. Due to the electromagnetic interaction of the loops and the slot, the resonance frequency of the latter moves to a lower value, as will be seen later; then, if this radiator covers a wider bandwidth, the required frequency can still be within the cutoff frequencies.

**Figure 4:**
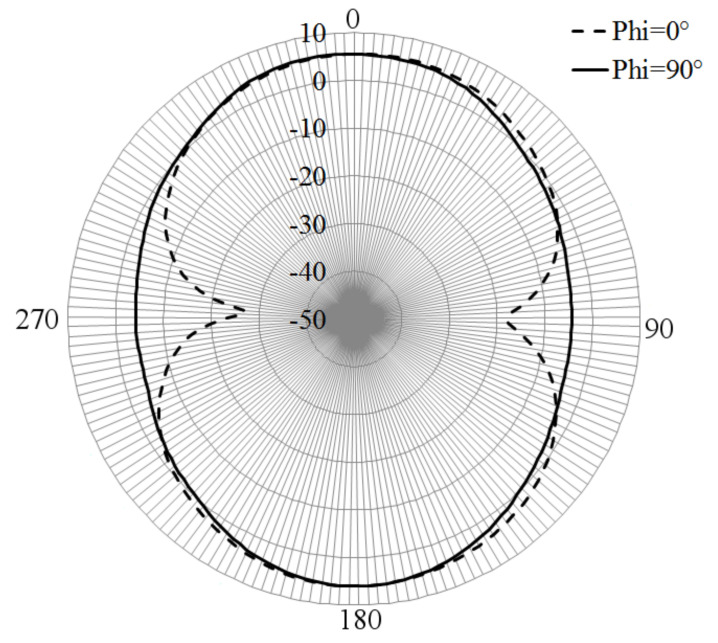
Simulated gain pattern of dumbbell-shaped slot.

The simulated radiation gain pattern of the dumbbell-shaped radiator is presented in Figure 4. From this point, the Z-X plane is the E-plane, and the X-Y plane is the H-plane for all the figures related to the gain pattern. It is observed that the gain pattern shows a bi-directive shape, presenting a gain around 5.5 dB at Theta = 0° and 180°, and −3 dB at Theta = 90° and 270°, for Phi = 0°. For the plane at Phi = 0°, the gain pattern shows deeper nulls at Theta = 90° and 270°.

**Figure 5:**
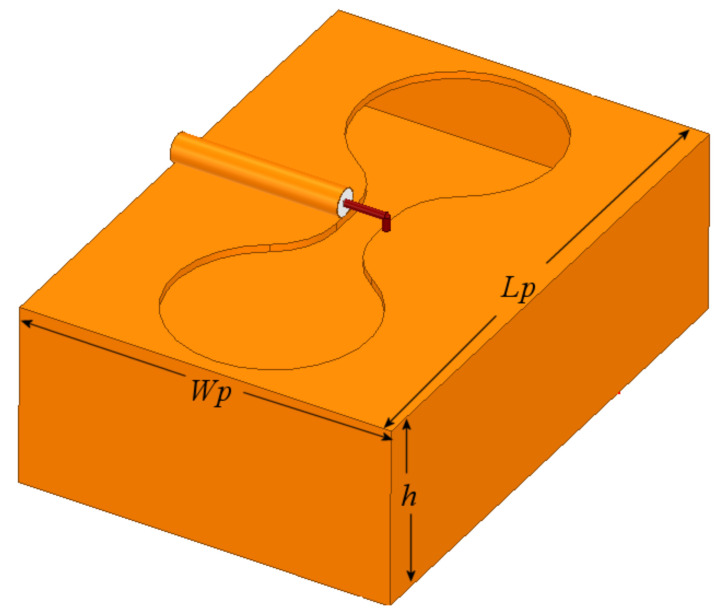
Dumbbell-shaped slot radiator with box reflector.

**Figure 6:**
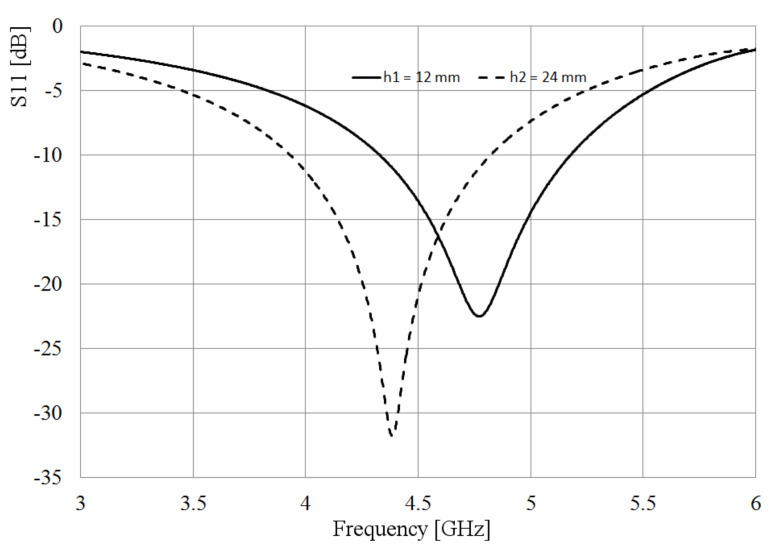
S11-parameter of the antenna for different box sizes.

**Figure 7:**
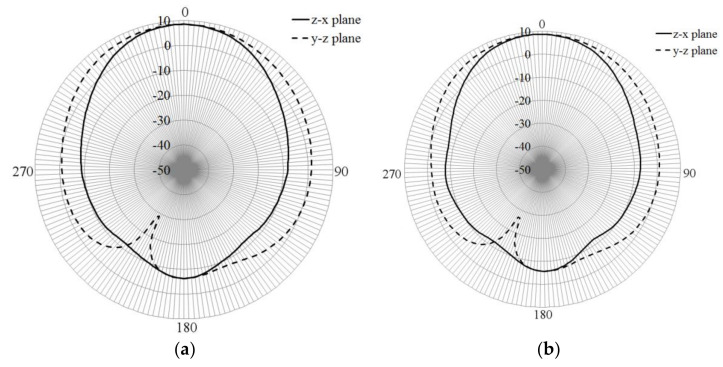
Simulated gain pattern of the dumbbell-shaped slot radiator with box reflector: (**a**) h1, (**b**) h2.

To modify the radiation gain pattern of the slot to a directive one, several techniques lead to the desired result. In this work, a metallic box is introduced not only to provide the directivity but also to confine the energy and point it out to the chosen direction. This box-reflector also allows reducing the magnitude of undesired lobes, simultaneously on the x and y axes (see Figure 1 for axes reference). The reflector dimensions were chosen considering a compromise such that the size is as small as possible without degrading the coupling and the gain of the antenna. Conserving the size of the metal sheet where the dumbbell-shaped slot was embedded, the effect of the depth, h, of the reflector was investigated with the help of HFSS. Figure 5 shows the configuration of the dumbbell-shaped slot and the reflector box. Figure 6 and Figure 7 show the results obtained for two values of h, h1 = 12 mm, and h2 = 24 mm.

From Figure 6, it can be found that with h = 24 mm, the port coupling is not degraded. Moreover, it is confirmed that the resonance frequency of the slot radiator depends on the finite dimensions of the metal sheet where it was inserted. This fact is observed when the box is smaller since the frequency shifts to 4.77 GHz, 230 MHz lower than the frequency of the slot without a reflector. Alternatively, when the box is bigger, the resonant frequency shifts down to 4.38 GHz, 620 MHz lower than the original antenna, but the port matching and the bandwidth are bigger.

The compromise of selecting the size of the box should consider the overall size of the entire antenna, the port matching required, and finally, the advantage or disadvantage of shifting the resonant frequency to a small or big frequency interval. The latter is important to be taken into account since, as will be observed, the extra resonances added by using loops can overlap the resonance of the slot. Moreover, in Figure 6, it is observed the advantage of performing a wide bandwidth. The slot was originally designed to resonate at 5 GHz; however, when the box is built beneath the slot, the resonance shifts down, and 5 GHz is still in the bandwidth when h = 12 mm.

On the other hand, Figure 7 displays the gain patterns for the two values of h, and in both cases, it is observed that the directivity is kept, and the gain is close to 9 dB.

As observed in Figure 7, both box sizes perform adequately to make the radiator directive. The gain for both heights is close to 9 dB, and the front-to-back lobe ratio is around 15 dB. The beamwidth for both cases is around 80°. Then, regarding the gain pattern, both boxes perform similarly, but as explained above, the bigger box offers better bandwidth and port matching. In the case that the frequency of 5 GHz is strictly required, a slightly reduction of the slot length can be considered.

Having in mind the previous results, the reflector/box with dimensions 64 × 49 × 24 mm^3^ is employed for the final design. The next step in the process is the integration of the rectangular loop resonators into the slot radiator to obtain the desired frequencies, besides the one given by the slot.

*B*. *Design of rectangular loop radiators*

**Figure 8:**
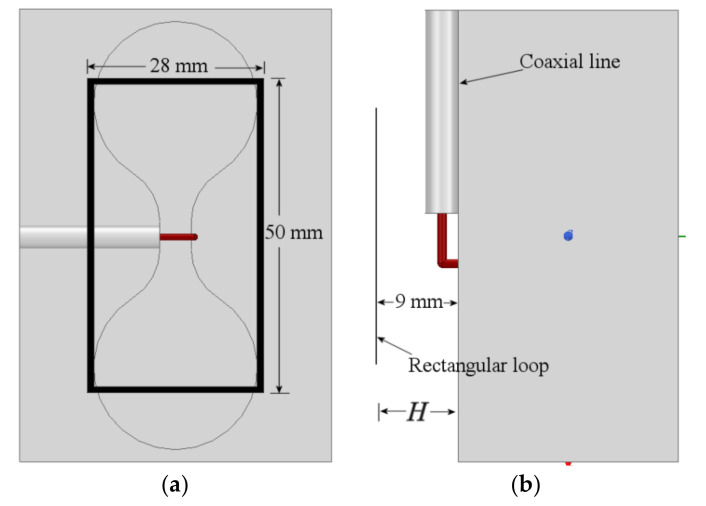
Rectangular loop and dumbbell slot antenna: (**a**) top-view, (**b**) side-view.

Antenna loops present a resonance to a specific frequency when their perimeter is close a wavelength. For self-resonance, each radiator must have a perimeter close to a wavelength. For example, for a 1.95 GHz resonant frequency, the perimeter of the loop is 154 mm. The implementation of this ring is presented in Figure 8. The rectangular loop is perfectly center on the dumbbell-shaped slot. An adjustment from 154 mm to 156 mm, considering the outer edge of the ring, was done to shift-down the resonance to 1.95 GHz. The loop conductor width is set to 1 mm. The separation H between the box surface and the ring, as will be shown later, plays an important role in the port matching at the different frequencies. In the initial design, it is set to 9 mm.

**Figure 9:**
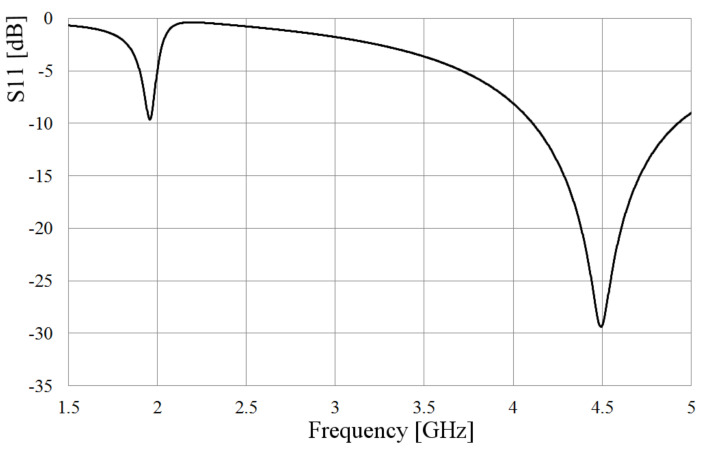
Simulated S11 parameter of the loop-slot antenna.

The simulated S11 parameter of the assembly shown in Figure 8 is presented in Figure 9. As observed in this figure, the new resonance is 1.95 GHz, as calculated, and the resonance of the slot is preserved around 4.5 GHz. At 5 GH, the port matching is around −9 dB. Although both desired frequencies are mismatched, showing a value slightly bigger than −10 dB, it is a result that will be improved later.

**Figure 10:**
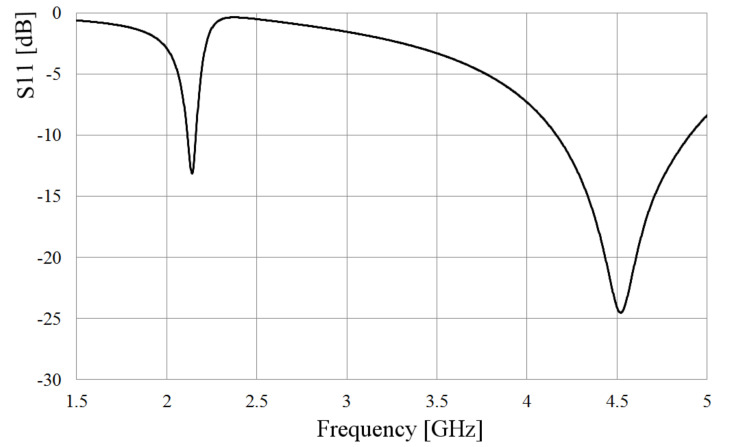
Simulated S11 parameter of the second loop-slot antenna.

Now, following the procedure for the first ring, a second one is calculated to resonate at 2.14 GHz. The perimeter of the loop is 140 mm, but after a simulation process, this length was changed to 144 mm to adjust the resonance. The separation between the loop and the box is also kept to 9 mm. The simulated S11 parameter is presented in Figure 10.

**Figure 11:**
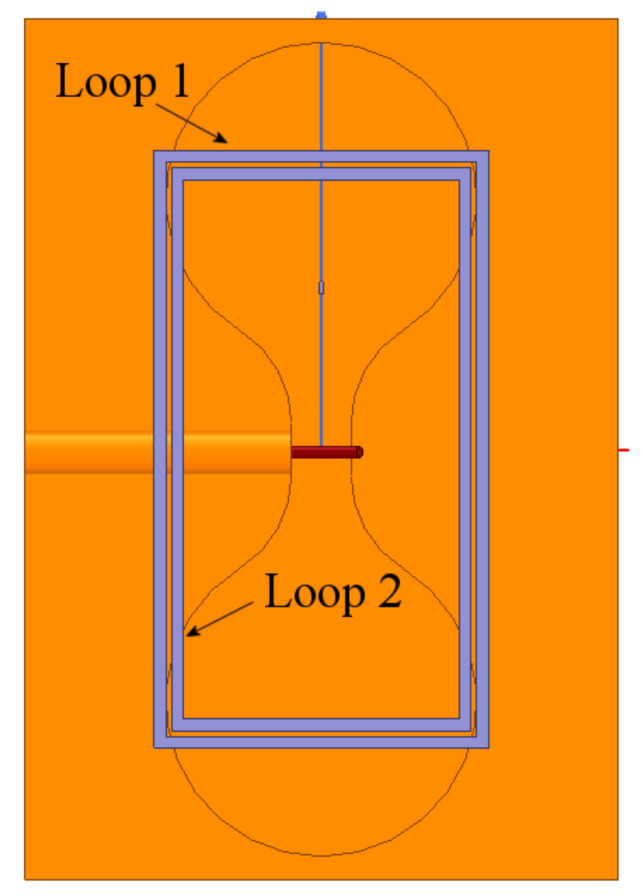
Loops 1 and 2 set along over the dumbbell-shaped slot and box.

As observed, the resonant frequency is obtained at 2.14 GHz, and the slot’s resonance is maintained at 4.5 GHz. Now, both loops are integrated into the radiating slot, as presented in Figure 11. This fact will lead to mutual electromagnetic coupling, and this coupling changes the resonance of both loops, so if an exact resonance is desired, a second process of tuning of the ring size must be carried out.

**Figure 12:**
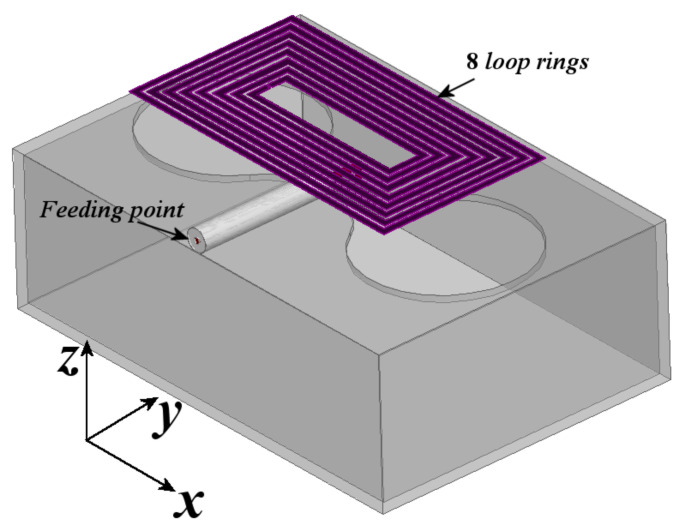
Proposed configuration of 8 rectangular loops combined with dumbbell-shaped slot radiator and box reflector.

The simulated S11 parameter of the slot with two loops is presented in Figure 12, and as predicted, the resonances of both loops are slightly shifted to lower frequencies. This shifting is due to the mutual loading on both rings caused by the electromagnetic coupling between the structures. This change in the resonant frequency can be approximated by calculating the associated extra-inductance generated between the two electromagnetic structures. The adjustment of the required frequencies is obtained with HFSS, by using a parametric analysis.

To provide more resonant bands, just as a design example, 6 more rectangular rings were introduced to the structure shown in Figure 11, and the resulting array is presented in Figure 12.

The configuration in Figure 12 shows that each new loop is concentric to the first two and separated by 0.5 mm. Table 1 shows the dimensions of each loop radiator.

**Figure 13:**
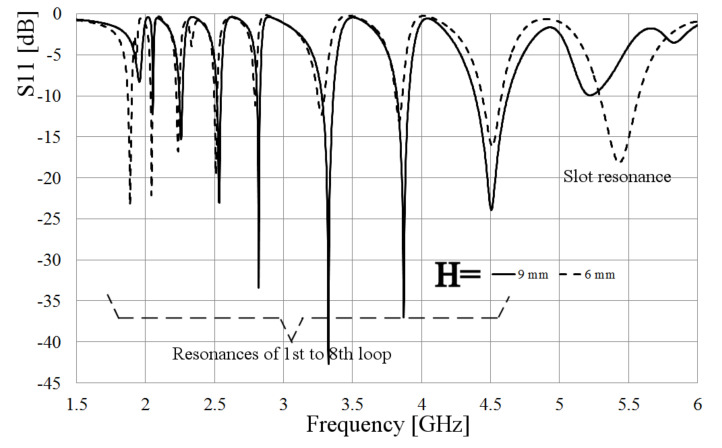
Comparison of simulated S11 parameter of the loop-slot array at different distances between them.

The simulated S11 parameter for the array is presented in Figure 13. In these results, the separation between the rings and the sheet of metal where the slot was engraved takes two values: 9 mm and 6 mm. It is noteworthy that several separations were simulated, but these two values were selected to explain the effects of this parameter in the antenna performance. As a result, and according to Figure 13, the smallest value is taken to match the port at all the resonances. This outcome allows to emphasize the coupling for one or more frequencies of interest by tuning the variable H. Recalling the results in Figure 9, the resonance of the biggest ring presents a mismatching; then, getting the rings closer to the box, the electromagnetic coupling to the slot is increased, and as a result, the port coupling at the lowest resonance changed from −8 dB to −22 dB.

From Figure 13, it is also observed that the first resonance shifts slightly to lower frequencies, and this is due to the increasing on the magnetic coupling, given by the interaction of the other rings and a closer approximation to the slot. This results in a bigger associated extra-inductance. Another difference that must be pointed out is the resonance obtained by the slot. In Figure 13, it is shown that the last resonance is around 5.5 GHz, showing an increase in the resonance, which is contrary to the effect presented in previous results. This is explained due to the covered area made by the rings over the slot, reducing its effective area and making it electrically smaller. As a result, this resonance is obtained at a higher frequency.

**Figure 14:**
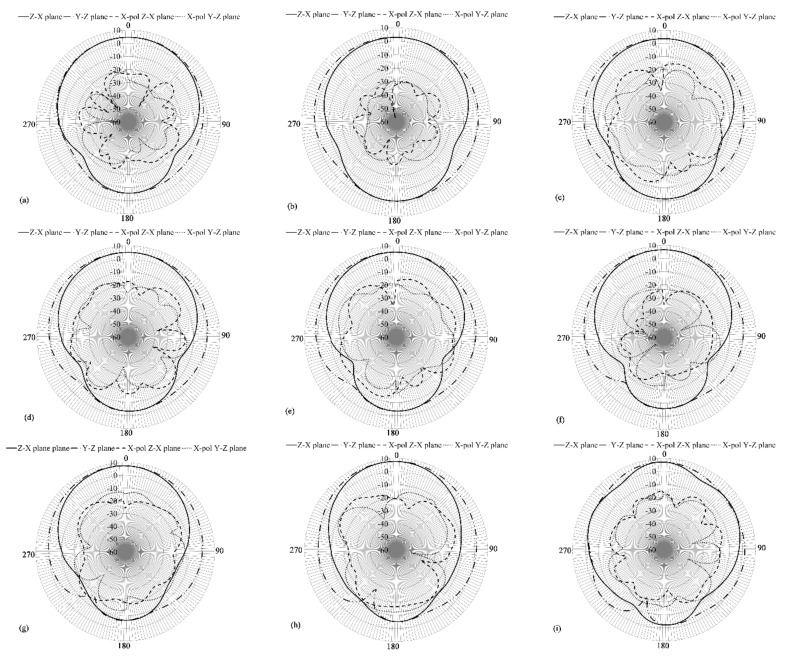
Simulated gain pattern of the proposed array, (**a**) 1.88 GHz, (**b**) 2 GHz, (**c**) 2.2 GHz, (**d**) 2.5 GHz, (**e**) 2.8 GHz, (**f**) 3.2 GHz, (**g**) 3.8 GHz, (**h**) 4.5 GHz, and (**i**) 5.5 GHz.

Afterward, Figure 14 shows the gain patterns at different frequencies at the y-z and z-x planes. From this figure, it is observed that the gain pattern keeps the directivity with gains varying from 3.5 dB, at lower frequencies, to 8 dB at higher frequencies. The front-to-back lobe ratio goes from 10 dB to 15 dB, and the cross-polarization levels on both planes are below 20 to 30 dB compared to the co-polarization pattern in all resonances. Once the antenna is designed, the next step is the construction and characterization of the prototype.

## 3. Measured Results

**Figure 15:**
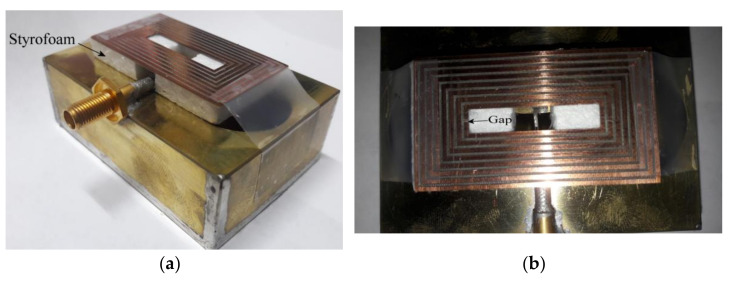
Prototype: (**a**) isometric view, (**b**) top view of rectangular rings.

Due to some problems found in the building process, the rings were made over a substrate with dielectric permittivity of 2.2 and thickness of 1.27 mm; otherwise, the rings were not rigid enough to maintain the strength. Because of the interaction of the substrate, the resonances obtained in the previous section were shifted to lower frequencies, and this is due to the increment in the associated capacitance given by the permittivity of the substrate. However, the phenomena explained above is preserved and for demonstration, an eight ring with dumbbell-slot is built. Moreover, to exemplify the frequency reconfiguration possibility, the smallest ring is split with a gap to avoid its resonance. The prototype is presented in Figure 15.

**Figure 16:**
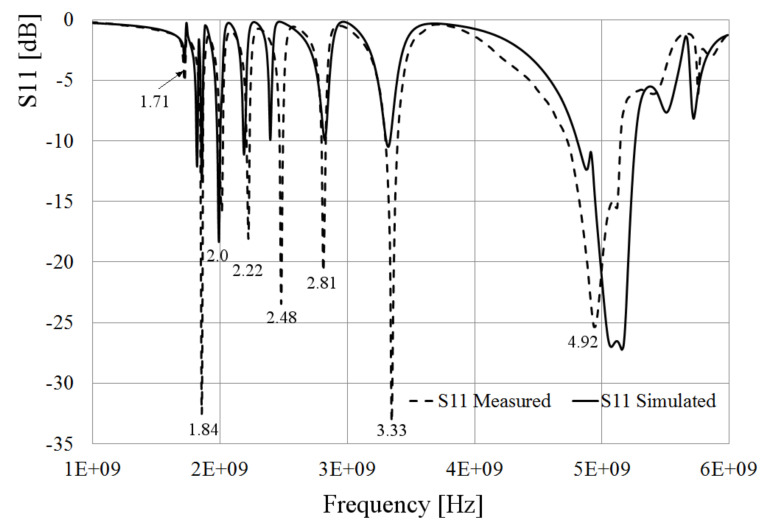
Comparison of simulated and measured S11 parameter.

As observed in Figure 15a, the rings etched on the substrate are supported by a layer of Styrofoam which permittivity is close to 1. Figure 15b presents the top view of the prototype where the gap on the smallest loop is pointed out. A comparison of the simulated and the measured S11 parameter is presented in Figure 16. In this case, the effect of the substrate is included in the simulation process.

From Figure 16, it is observed that there are 8 resonances, 7 of them derived from the 8 loops, taking into account that the smallest one is in an open circuit state; and then, its resonance does not arise. This process can be used in a future configuration to avoid certain resonances by a frequency control structure. On the other hand, the highest resonance is given by the dumbbell slot. Another important issue that must be pointed out is the mismatching suffered at the lowest resonance frequency. This mismatching occurs because of the interaction of the substrate on the biggest ring, changing its impedance and reducing the port coupling to −5 dB, approximately. This drawback can be fixed by tuning the separation of the ring itself from the slot, a process that was demonstrated by simulations in the previous section.

**Figure 17:**
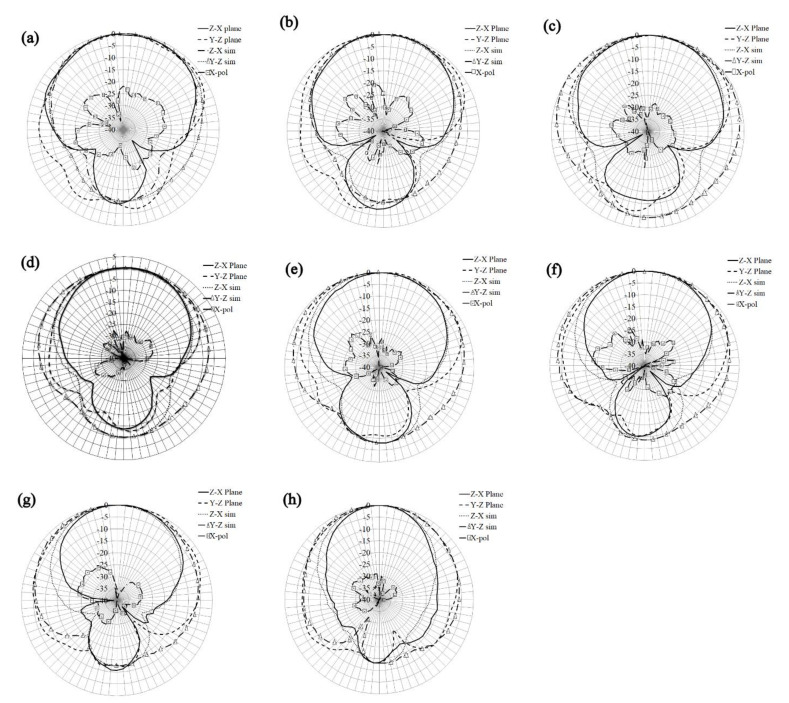
Measured gain patters at (**a**) 1.71 GHz, (**b**) 1.84 GHz, (**c**) 2 GHz, (**d**) 2.22 GHz, (**e**) 2.48GHz, (**f**) 2.81 GHz, (**g**) 3.33 GHz and (**h**) 4.92 GHz.

Next, the gain patterns of the antenna on the z-y Plane and the y-z Plane were measured, and the normalized results are plotted in Figure 17. Those results are compared to simulated co-pol results and measured cross-pol levels in the z-x plane, which is at least 20 dB below the co-pol pattern. To better understand the plots, the y-z plane cross-pol was omitted, since these results present similar levels to the z-x plane.

From Figure 17, the directive gain pattern is kept at all resonance frequencies, reaching a front to back lobe ratio of at least 10 dB. The beamwidth is around 60° at all frequencies, except for the last one which presents approximately 40° on this parameter on the z-x plane, showing a great convergence to simulated results. The measured gain and the radiation efficiency at each resonance are presented in Table 2. The gain was measured in a full anechoic chamber with a calibrated horn antenna, using the free space loss equation. The prototype and the horn antenna were connected to the measurement equipment through steady-phase cables, and the efficiency was measured by using the Wheeler Cap Method [21].

From Table 2, as expected, the gain goes from a lower to a higher value, achieving around 4 dB to 8 dB from the lowest resonance to the highest one. The efficiency also increases as the frequency does, as well as the bandwidth which is good enough for communications applications.

## 4. Main Results

The proposed configuration presents a combination of a loop array, excited by a wideband slot, which provides multiple band operation. The bans can be selected by designing the loop with a wavelength perimeter at the desired frequency. This configuration presents good results since the multiband can be controlled. The configuration allows multiple resonances; in this paper, 9 resonances can be obtained, but the number can be chosen to a lower or higher value according to the necessities. The configuration does not allow any other kind of resonator to be employed, for example monopoles instead of loops, since the resonances are not excited, and no multiband operation is obtained. The same phenomenon happens when other radiators are employed, such as slots of patch antennas; then, the use of loops is a very valid and innovative way to obtain the multiple resonances with lower cost, small profile and ease of build. The gain pattern can be adjusted from a bidirectional to directive shaped by introducing the box-reflector. For this reason, the antenna becomes more versatile than those given in state-of-the-art publications, such as [22], where a patch antenna is modified and introduced to a metamaterial to obtain multiple resonances, and the antenna is low profile; however, just a few resonances are obtained, and these are due mainly to the excitation of higher propagation modes, besides the difficulty of designing the metamaterial. In [23], a multiband antenna is presented; however, this antenna does not show multiple bands at the same time, making it just tunable. In [24], a compact multiband antenna is also presented, but only a small number of resonances are obtained. In [15], a very similar configuration is presented, where the excitation line is implemented with a microstrip line. However, the electromagnetic coupling between the rings and the line is also made through a slot structure, each ring needing an independent slot. It means that for *n* resonances, *n* slots are required. In addition, in [15] the resonances are originated exclusively by the rings, while in this work, an extra resonance is given by the slot. Table 3 presents a comparison of the structure given in [15] and the present work.

## 5. Conclusions

In this paper, a multiband antenna is presented, which is mainly composed of a set of rectangular concentric loops designed to resonate at different frequencies and excited by a radiating wideband slot. The slot is shaped to obtain a wider bandwidth, applying the Kraus technique in which the transitions are smoothed, reaching a circular dumbbell-shaped structure. This slot shape is needed to overcome any frequency shifting due to a reactive load generated when the loops are introduced and positioned above the slot. That means, when the resonance of the slot changes, the required frequency can be still between the operational bandwidth. The characterization of the antenna shows that the directivity and the ratio of the main lobe to the back one are conserved high for all resonance frequencies, as well as gains in the range of 4 to 8 dB, achieving also a very low cross-polarization level. It was demonstrated that the antenna can perform as a directive element by including a box-shaped reflector or quasi-omnidirectional one if the reflector is not employed, making it versatile compared to other prototypes. Moreover, it is not difficult to manufacture, with low-cost, low profile (without reflector), and compatibility with different kinds of communications systems.

## Figures and Tables

**Table 1:** Dimensions of rectangular loops in the array.

Number of Loop	Width [mm]	Length [mm]
1	28	50
2	25	47
3	22	44
4	19	41
5	16	38
6	13	34.5
7	10	31
8	7	28

**Table 2:** Gain and radiation efficiency.

Frequency [GHz]	Gain [dB]	Efficiency [%]	BW [MHz]
1.71	3.8	86.5	NA *
1.84	4.6	88	24
2.0	4.0	89.5	36
2.22	5.2	91.5	37
2.48	5.3	92	49
2.81	6.3	95	49
3.33	6.7	95	98
4.92	8.0	97	440

* NA: Not available.

**Table 3:** Comparison of [15] and this work.

Parameter	[15]	This Work
Number of resonances	3	8
Size	150 mm × 150 mm (base)	64 mm × 49 mm
Feeding technique	Microstrip with 3 slots	Coax-line with 1 slot
Higher modes excited	Yes	No
Gain [dB]	5 to 6	4 to 8
Resonance Bandwidth [MHz]	NA *	>20

* NA: Not available.

## Data Availability

Not applicable.
